# Masticatory Function in Individuals with Temporomandibular Disorders: A Systematic Review and Meta-Analysis

**DOI:** 10.3390/life13020472

**Published:** 2023-02-08

**Authors:** Vanessa Marcelino, Solène De Rovere, Maria Paço, Maria Gonçalves, Sandra Marcelino, António Sérgio Guimarães, Teresa Pinho

**Affiliations:** 1 UNIPRO—Oral Pathology and Rehabilitation Research Unit, University Institute of Health Sciences (IUCS), Cooperativa de Ensino Superior Politécnico e Universitário (CESPU), 4585-116 Gandra, Portugal; 2 TOXRUN—Toxicology Research Unit, University Institute of Health Sciences (IUCS), CESPU, CRL, 4585-116 Gandra, Portugal; 3 Centro de Investigação de Montanha (CIMO), Instituto Politécnico de Bragança, Campus de Santa Apolónia, 5300-253 Bragança, Portugal; 4 Laboratory of Neuroimmune Interface of Pain Research, Faculdade São Leopoldo Mandic, Campinas 13045-755, Brazil; 5 IBMC—Instituto Biologia Molecular e Celular, i3S—Instituto de Inovação e Investigação em Saúde, Universidade do Porto, 4200-135 Porto, Portugal

**Keywords:** temporomandibular disorders, electromyography, mastication, masticatory efficiency, chewing, bite force

## Abstract

The literature search was performed according to the Preferred Reporting Items for Systematic Reviews and Meta-Analysis (PRISMA) protocol in the PubMed, Cochrane Library, LILACS, EBSCO, Scielo, between 2012 and 2022. The methodological quality was assessed by using the Newcastle–Ottawa Study Quality Assessment Scale. Mean differences and 95% confidence intervals were calculated and combined in meta-analyses. A total of 1202 participants were included in this systematic review (690 with TMD; 512 without TMD), with 22 articles being included in the qualitative analysis. Only three studies enabled the comparative analysis of the results. Ten articles showed a high methodological quality and a low risk of bias, and twelve had a low methodological quality and an increased risk of bias. The meta-analysis showed that the differences between the intervention and control groups were not statistically significant for the percentage overlapping coefficient of the anterior temporal muscle, for the masseter, and for the torque coefficient. The parameters analyzed with the compound technique for chewing showed altered mandibular functions in individuals with TMD. With the EMG method, it was possible to suggest that TMD in adult individuals causes compensatory muscle behaviors, and several changes in the masticatory function were found.

## 1. Introduction

Temporomandibular disorders (TMDs) involve the pathologies of the orofacial region with neoplastic, traumatic, and/or musculoskeletal disorders [1]. TMD involves signs and symptoms such as joint and/or muscle pain, limited mandibular movement, altered masticatory muscle function, and joint noises [2]. The manifestation of one of these factors, or the combination of several, may adversely influence the performance of stomatognathic functions, namely chewing and swallowing [3]. Epidemiological studies indicate a high prevalence of TMD of approximately 31% in adults and the elderly population, with the most prevalent temporomandibular joint disorders (TMJs) being the disc displacement with reduction (DDwR) [4]. Some studies have shown that TMD affects more female individuals, i.e., in a ratio of 3 to 1 [5,6,7,8,9].

Pain is one of the most common symptoms described by TMD subjects [10]. The literature describes that these individuals suffer from masticatory function limitations [11] because their mandibular movements adapt in a conscious or unconscious attempt to avoid painful stimuli. Difficulty swallowing hard foods and tiredness after chewing have been reported in TMD patients [4,12]. Changes in the masticatory muscles’ recruitment and an increased asymmetry between the right and left sides were also reported when comparing individuals suffering from TMD to asymptomatic ones [3]. As chewing is one of the essential functions of the stomatognathic system [13], it is critical to understand the functional and clinical changes related to TMD, as well as their consequences. Some TMJ can influence the normal functioning of mastication, altering its type and pattern [14,15,16]. These alterations can cause numerous problems in need of treatment from several clinical specialists, such as speech therapists, physiotherapists, and dentists, among others [17,18,19,20]. Often, eating limitations match the self-reports of jaw pain, fatigue, or jaw noises during biting [21,22,23] and some physical examination findings, such as decreased activation, strength, or endurance of the masticatory muscles and/or diminished force production [23]. A recent study revealed that unilateral TMD involves an alteration of the preferred chewing side, being also accompanied by TMJ remodeling [24]. Furthermore, the correlation between TMD and mandibular kinematics range of movements parameters, such as maximum mouth opening, lateralization, and maximum protrusion/retrusion, has been described, and it was found that values tend to be increased or decreased depending on the type of TMD [19].

Surface electromyography (sEMG) is a technique that contributes to a better knowledge of muscle physiology and assists in the differential diagnosis and monitoring of TMD [25]. This diagnostic tool can assess the behavior of muscles intervening in the TMJ at rest and during human jaw motion [26]. Surface electromyography (sEMG) is a reliable and valid tool to evaluate muscle activity and, therefore, may be useful in the evaluation of TMD patients. sEMG detects electrical potentials and, on this account, may conceivably be employed in TMD diagnosis [27]. A chewing compound is considered one of the most valuable test materials to evaluate the ability to chew and assess the parameters of masticatory efficiency. It has stability in quality and uniformity as a manufactured product and can be produced on a large scale [28]. Masticatory efficiency (ME) can be defined as the ability to fragment food within a given time interval and can be measured by an individual’s ability to fractionate natural or artificial foods [29].

It is important for professionals to know the clinical manifestations of TMD and to understand the influence of TMD on the individual’s habitual chewing. Surface electromyography (sEMG) is a diagnostic tool that ensures reliable and valid evaluation of muscle activity. It detects electrical potentials and, on this account, may conceivably be employed in TMD diagnosis [30]. Collecting accurate data on the temporomandibular complex is important to create and adapt the treatment to each case, evaluating the previously mentioned variables. This study aimed to summarize the scientific evidence regarding the assessment of masticatory function in adult individuals with TMD, using two different techniques, namely chewing material and electromyography, as well as evaluate the methodological quality of the included studies and to perform a meta-analysis.

## 2. Materials and Methods

The literature search was performed according to the Preferred Reporting Items for Systematic Reviews and Meta-Analysis (PRISMA) protocol. The research question was defined as follows: “Do individuals with TMD present changes in masticatory function, evaluated through a chewing compound or an electromyographic technique when compared with individuals without TMD?” The scientific question was structured according to the acronym PECO (Population, Exposure, Comparison, and Outcome; see Table 1), establishing the primary inclusion criteria for the studies that were selected a priori. No language limitation was set.

The exclusion criteria were set as follows: (1) the presence of with systemic diseases, degenerative diseases or neuromuscular disorders; (2) unavailable full text; (3) TMD diagnostic tools other than RDC-TMD or DC-TMD; (4) participants with malocclusions; and (5) participants wearing dental prostheses.

### 2.1. Information Sources and Search Strategies

The search was conducted in different databases (Cochrane Library, PubMed, LILACS, EBSC, and SciELO) to include all relevant literature on this topic. The search strategy was established before starting the database query. A register of unpublished or in-progress studies, called “grey literature” (ClinicalTrials.gov), assessed on the 16 June 2022, was also consulted to minimize publication bias. The search strategy was based on the combination of medical terms (Mesh) and keywords relating to the following concepts: “Temporomandibular disorders, electromyography, mastication, masticatory efficiency, chewing, bite force.” The complete search strategy is available in Appendix A, Appendix B, Appendix C, Appendix D, Appendix E, Appendix F and Appendix G.

### 2.2. Study Selection

**Figure 1:**
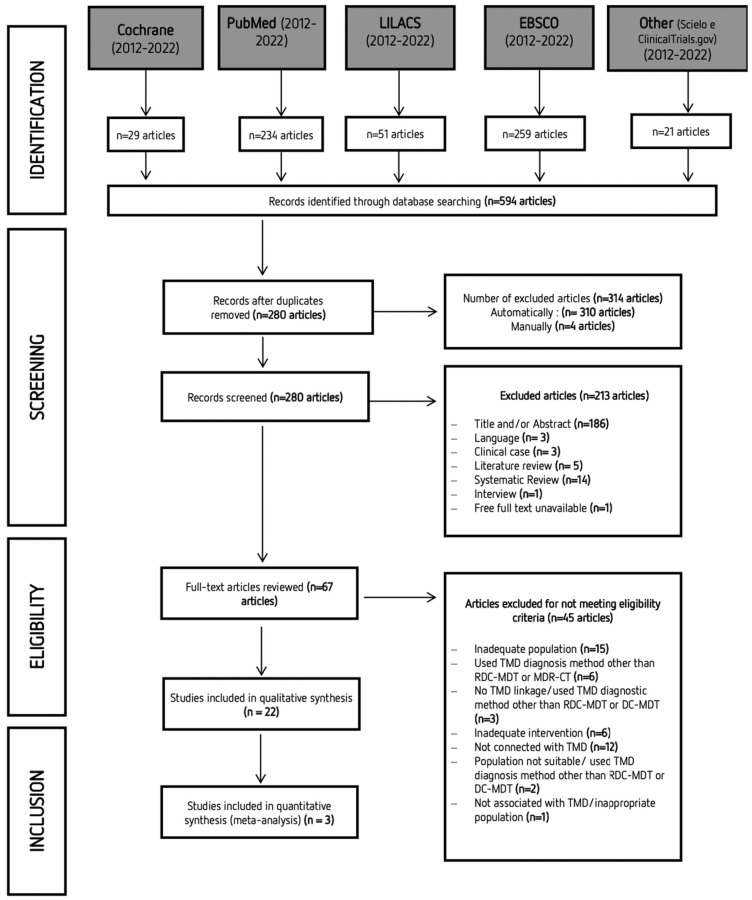
PRISMA (Preferred Reporting Items for Systematic Reviews and Meta-Analyses) flow diagram of the search strategy and obtained results.

A systematic search was performed with articles published between 2012 and 2022. The last online search was performed on the 16 June 2022. However, because different databases were explored, it is frequent to find duplicate articles. Study selection was initially carried out independently by two researchers (S and VM) via title and abstract reading. Studies that did not complete the eligibility criteria were discarded. In the second phase of this selection, the same investigators independently applied the same eligibility criteria to the full texts, compared decisions, and resolved differences by discussion and consultation with experienced investigators (TP and MP) whenever consensus could not be reached. The process of identifying, screening, and excluding studies followed the strategy shown in Figure 1. Most of the studies that Mendeley did not identify as duplicates had minor changes in the title or the original language.

### 2.3. Data Extraction

Data extraction is displayed in Table 2, where information such as study design, sample, age, diagnostic method, chewing evaluation method, variables analyzed, and results is shown. Data extraction was carried out independently by two researchers (S and VM); any disagreement was solved by discussion, and when necessary, a third author (TP) was consulted.

### 2.4. Data Analysis

The meta-analysis was performed by using the Review Manager software, version 5.4. Forest plots were created to present the combined estimates for which two or more studies had similar EMG signal collection and processing methods. Statistical heterogeneity among studies was assessed by using the I^2^ test. Forest plots were created to present the combined estimates for which two or more studies had similar EMG signal collection and processing methods. Statistical heterogeneity among studies was assessed by using the I^2^ test. A value of I^2^ > 50% is considered to indicate large heterogeneity. In the presence of large heterogeneity, a random-effects model was used; otherwise, a fixed-effects model was used. The result was considered statistically significant when the *p*-value was <0.05, or if the 95% CI (confidence interval) about the mean differences did not cross 0 (zero).

### 2.5. Risk of Bias in the Studies

This systematic review used the Newcastle–Ottawa scale (NOS) to assess the methodological quality of the included studies. NOS presents 3 parameters: selection, comparability, and outcomes. A study can be given a maximum of one star for each numbered item in the selection and outcomes. A maximum of two stars can be given for comparability, with a maximum score of 9, indicating the highest quality studies [53]. Two researchers (S and VM) did this assessment independently and in duplicate. Again, any disagreements were solved through discussion with experienced researchers (TP and MP).

## 3. Results

### 3.1. Studies Selection

After completing the first stage of search in databases, a total of 596 studies were obtained. After eliminating duplicate articles, the remaining 280 articles were assessed via title and abstract reading. Only 67 articles were retained for full-text reading. Twenty-two articles were included for the qualitative analysis. The characteristics of the selected studies are summarized in Table 3. A total of 1202 participants were included in the studies that were analyzed in this systematic review (690 with TMD; 512 without TMD).

### 3.2. Study Characteristics

Fifteen of the selected articles used the RDC/TMD protocol to diagnose TMD [31,34,37,38,39,40,41,42,43,44,45,50,54,55,56], and the seven others used DC/TMD [32,33,35,36,48,51,52]. Mapelli et al. (2016) [38] was the only study that presented, within the group defined as “with TMD,” a subdivision according to the pathology’s severity: moderate and severe. The publication date criterium revealed six articles > 6 years [31,42,43,50,54,55], eight articles between 3 and 6 years [34,38,39,40,41,44,45,48], and eight from the last 3 years [32,33,35,36,37,51,52,56]. Studies using electromyography to assess chewing function are more recent [32,35,37,51] than those using chewing gums [31,32,54,55]. All articles are available in English, and the authors’ country of origin and the sample are usually the same. We found a significant predominance of studies from Brazil (*n* = 16) [3,31,33,34,35,36,38,39,40,41,42,43,44,50,52,56]. Our list of countries includes Italy [32,51], China [45], Poland [48] and Turkey [37,54]. Regarding the protocol used to assess masticatory function, all studies performed static and/or dynamic tests in MVC (Maximum Voluntary Contraction), and six also evaluated the stomatognathic system during mandibular rest [34,36,41,48,50,52]. Concerning gender, thirteen studies only included female subjects [31,32,33,34,39,40,41,42,43,48,51,52,55]. Regarding the sample size of the studied populations, of the twenty-two selected articles, two studies [40,43] presented small samples (*n* = 22), contrasting with the two articles [32,39], which presented a higher total sample (*n* = 104). The other studies have intermediate sample sizes, ranging between 25 [37] and 100 [48] individuals.

### 3.3. Risk of Bias in the Studies

The risk of bias in the included studies and the description of the aspects contained in the NOS scale are summarized in Table 4. When analyzing the risk of bias, it was shown that ten studies presented high methodological quality and low risk of bias [32,33,34,35,41,50,51,52,55,56], and twelve were classified as being of low quality [31,36,37,38,39,40,42,43,44,45,48,54].

### 3.4. Masticatory Function

The masticatory function was evaluated by different aspects/instruments in the included studies. Some studies evaluated the muscular activity through the electrical intensity of masseter and temporal muscles [31,33,34,35,36,37,38,39,41,43,44,45,48,50,51,52,55], the symmetry [31,32,33,38,42,51,52,55], and the synergy [32,38,51,52], and others evaluated the masticatory efficiency [44,54]. Besides the EMG and masticatory compound, the included studies described the masticatory function with a computerized mandibular scanner in two studies [36,54], a digital dynamometer in two studies [40,55]; in one, the authors used a pressure transducer [50]; in one, a bite force transducer [46]; in another one, an ultrasonography [50]; in one study, they used a sonography [36]; in one, a vibraphone [37]; in one, a computerized digital occlusal with T-Scan III [37]; and at last, in one study, a mandible kinesiograph [54].

#### 3.4.1. Muscle Activity in MVC

Twelve studies [31,34,35,36,37,38,39,41,45,48,51,52] compared the muscular intensity between TMD and control subjects. An increase in electrical intensity was found in two of them [45,51]. In both, the TMD groups presented greater values of muscular activation than the control group [45]. In addition, in one of those [51], higher activation of masseter muscle was only observed in TMD patients in comparison with the control group. However, seven studies found that TMD leads to reduced values of muscular activation for both muscles [35,36,38,39,41,48,52], happening more frequently in the temporal [48] or more in the masseter [39]. In the other three studies [31,34,37], no relevant differences were found between the studied groups. Five articles [31,32,38,51,52] also evaluated muscle force symmetry/coordination, where three of them showed statistical differences between both groups [31,38,52], revealing that subjects with TMD presented greater asymmetry of both muscles [31] or specifically in one of them (temporalis [38] or masseter [52]). They also presented significantly larger unbalanced contractile activities of the contralateral masseter and temporal muscles. Four studies [32,38,51,52] assessed muscle synergy, and all of them showed that TMD leads to greater muscle activity asynergy between the pairs of muscles (masseter or temporal muscles). Four studies [39,40,41,45] evaluated the median frequency during MVC, and three [39,40,45] of those studies did not find any statistical difference between TMD and control groups; however, the fourth study [41] found a reduced frequency. Only one study [48] evaluated the frequency index with the aid of EMG during MVC and found a diminution of these parameters in the sample with TMD in comparison with the healthy individuals.

#### 3.4.2. Muscle Activity at Rest

Four studies [36,48,50,52] assessed the electrical intensity at rest, but only one [48] showed increased muscular activity in both muscles, more in the masseter than in the temporalis, compared to the control group. One study also evaluated the muscle force symmetry/coordination [52] and showed a decrease in the symmetry index only for the temporal muscle.

#### 3.4.3. Muscle Activity in Dynamic

Six studies [33,37,38,43,44,55] compared the electrical intensity between TMD and control groups. A statistical difference was found in two of them [33,44], where the TMD group presented greater electrical intensity values than the control group, specifically during the agonist phase in non-habitual chewing [33]. However, during the agonist phase in habitual chewing, TMD leads to decreased muscular activation values for the masseter muscle [33]. The other studies [37,38,43,55] did not find any relevant differences between the studied groups. Of the two studies that evaluated the “global activity” and “activity/cycle of chewing” [38,55], only one found significantly higher values of these parameters in the TMD group [55]. Five studies [32,33,38,42,55] also evaluated muscle symmetry/coordination, and all showed that TMD leads to a more asymmetrical activity; one study specified that it happened only in the temporal muscle [42], and another one only during the habitual chewing [33]. Three studies [33,35,42] assessed muscle synergy, but only two observed a decrease in the synergy between the masseter and temporalis muscles of both sides in the TMD group. Moreover, the TMD group showed a greater relative energy than the control group [33,35]. One article [38] measured the Functional Index (FI) during chewing and found that the global functioning condition of the masticatory system decreased to form the control group to the TMD group. In one study, chewing was examined by using the Functional Index (FI), and it was found that the TMD group’s overall masticatory system function was lower than the one of the control group. The TMD group showed a longer chewing stroke duration than the control group [35].

### 3.5. Results by Chewing Analysis

The chewing process was analyzed in six studies [31,35,44,54,55,56]. From these, one tested chewing through capsule with fuscin [44], and five used cookies [31,35,55,56] or gelatin cubes [54].

Chewing analysis using chewing compound:

OMES-Score: Four studies [31,35,55,56] agreed with the fact that subjects with TMD have greater chewing difficulty than the control group, presenting a decreased OMES-score.

Chewing stroke duration and the number of stokes: These parameters were increased in the TMD group in comparison to the control group [44].

Frequency index: Two studies [38,51] evaluated the frequency of chewing and did not suggest any statistical difference between TMD and control groups.

Masticatory efficiency: The two studies [44,54] that studied “masticatory efficiency” found contradictory data; one [44] concluded that TMD leads to an increase in masticatory efficiency, and the other [54] presented a decreased for the same parameter.

#### Meta-Analysis

**Figure 2:**
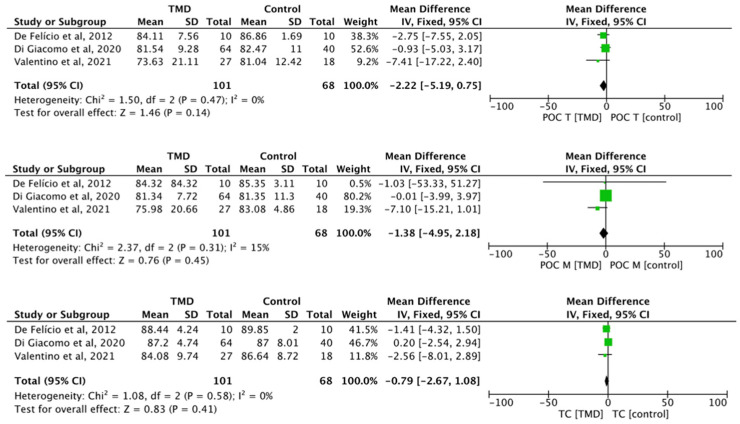
Forest plot of POC T, POC M, and TC of electromyographic signal with TMD compared to healthy controls. Values represent effect sizes (weighted mean differences) and 95% confidence intervals (CI). SD, standard deviation; I^2^, I-squared. Black diamond represents the overall effect estimate of the meta-analysis. Green squares represent each studies individual standardized mean difference (SMD) and the extending lines the confidence intervals [31,32,51].

The differences between TMD and control for the parameters’ percentage overlapping coefficient of the anterior temporal muscle (POC T) (MD −2.22, 95% CI −5.19 to 0.75; I^2^ = 0%; *p* = 0.47; *n* = 3), percentage overlapping coefficient of the masseter muscle (POC M) (MD −1.38, 95% CI −4.95 to 2.18; I^2^ = 0%; *p* = 0.31; *n* = 3), and torque coefficient (TOC) (MD −0.79, 95% CI −2.67 to 1.08; I^2^ = 0%; *p* = 0.58; *n* = 3) did not present any statistical significance (Figure 2).

## 4. Discussion

To the best of the authors’ knowledge, this is the first systematic review that compares two assessment methods to analyze masticatory function in adult individuals with and without TMD. Many studies used sEMG to assess the functional status of the masticatory muscles in individuals with TMD [17,18,20,47,49,57,58,59,60,61,62,63]. However, fewer used chewing compounds for this analysis [31,35,44,54,55,56]. From the 22 included studies, 19 presented different degrees of association between TMD and chewing function [31,32,33,35,36,37,39,40,41,42,44,48,50,51,52,54,55,56]. When using the chewing compound method, one study found that TMD leads to an increase in the masticatory efficiency with the fuchsin capsules [44], whereas another one found the opposite result with a decreased masticatory efficiency with gelatin cubes [54]. Peroz et al. (2002) [64] reported that pain promotes a tendency to chew food more cautiously, thus obtaining smaller pieces, leading to an increased chewing time and, consequently, greater chewing efficiency. For this reason, we are unable to reach a conclusion regarding the direction of the impact on the masticatory efficiency, only that it seems to be influenced by the presence of TMD. Nonetheless, it can be suggested that, clinically, it may be useful to use the masticatory compound to highlight any chewing-pattern changes. For future studies, we may recommend a more careful description of the sample depending on the subgroups, allowing a better comparison. Homogenization of the type of masticatory compound is necessary, too, since it is known that masticatory behavior can be altered depending on the foods’ texture [65]. The sequence of a mastication cycle is constituted by a set of movements that occur from food ingestion all the way to swallowing it. Although the number of cycles required to chew the same type of food is relatively constant for the same individual, it has sizeable inter-individual variations [66]. With the chewing-gum method, we found that individuals with TMD exhibited an increase in the number of chewing cycles and the time required to perform a cycle compared to healthy individuals [44]. This does not mean that TMD individuals have better chewing function, but it is possible to suggest that the patient developed this adaptation to prevent pain exacerbation [67]. Nonetheless, when the frequency index was used, no alteration was found [38,51]. Authors should preferably use the masticatory frequency index because it expresses the normalization of the number of masticatory cycles in relation to the execution time, since this discrepancy could be avoided by using the same parameter [51].

Regarding the analysis through sEMG, six studies [33,37,38,43,44,55] evaluated the electrical intensity of muscles during chewing and showed discrepancies in the results. Four of them found no differences between groups, and the other two showed that the electrical intensity of mastication muscles in individuals with TMD is greater than the intensity found in the healthy control group [33,44]. This increased intensity may not be associated with greater muscle strength but rather with the recruitment of new motor units to compensate for any asymmetry [38] and thus, improving neuromuscular coordination that is needed for the masticatory movements [68]. These variations may be the result of the use of a different sEMG pattern or chewing evaluation technique. In fact, mastication patterns differ during masticatory activity [33], and the differences in muscular intensity between TMD subjects and healthy individuals are more evident when collected under guided conditions (unusual and unilateral chewing) [40]. All the studies showed that individuals with TMD entail impaired orofacial motor functions, which may be related to the asymmetry of muscle activity, inducing a change in the mandibular movement itself [69]. All the articles that studied the symmetry during dynamic setup showed that patients with TMD disorders presented an altered muscular contraction [32,33,38,42,55], being more asymmetric during masticatory activity, specifically in the temporal muscle [42] or only during the habitual chewing [33]. Two studies [55,56] found that TMD patients showed impairment of orofacial motor functions, with alterations in the recruitment of masseter and temporal muscles during chewing. Two studies [55,70] reported the importance of this topic, considering that the general population which presents any sign or symptom of TMD may have the chewing process affected [71]. The analysis of the muscular electrical activity during MVC did not show any differences between the control and the TMD groups in terms of activation of one or both muscles. It permitted researchers to identify if one muscle is more activated than the other. An increase in electrical intensity was found in two studies [45,51] where the TMD group presented greater values of muscular activation than the control group [45]. In addition, in the second article [51], only a higher activation of masseter muscle was observed in TMD patients in comparison with the control group. Some of the studies included in this systematic review and previous studies [35,36,38,39,41,48,52] found that TMD subjects have lower activities during MVC than normal subjects associated with a reduction of the number of masticatory cycles [48]. Those findings may be due to the lower efficiency of masticatory muscles [32] and the easy muscle fatigue [36,39,40,41,45]. The other studies [31,34,37] found no relevant differences between the studied groups. In future studies, it would be pertinent to select the sample through the TMD type presented by each individual in order to reduce discrepancies. In the analysis of symmetry, five studies have also evaluated muscle symmetry/coordination [31,32,38,51]; three of them showed larger unbalanced contractile activities of the contralateral masseter and temporal muscles between both groups [31,38,52], while the other two did not [32,51]. These symmetry changes may serve as an incentive for future research since these effects may suggest that individuals with TMD tend to present a functional alteration reflected in masticatory muscles coordination. However, a marked asynergia was noted in TMD subjects since a preponderance of activity was found in the masseter muscle [51] or the temporalis muscle [32,38]. Except for one article [48], all studies that evaluated the parameters in the mandibular resting position concluded that individuals with TMD showed no differences in muscular intensity compared with individuals without TMD [33,36,41,50]. Thus, using sEMG in the mandibular resting position does not prove to be the ideal method for TMD diagnosis. However, it is widely found in the literature in the stomatognathic system evaluation.

The different methods of sEMG signal capture, processing, and analysis constituted an important limiting factor for the comparative analysis of the results described in the articles selected in this systematic review. All articles described different sEMG capture and processing protocols. Such variations in methodological procedures hinder data analysis and indicate the need for a standardized protocol regarding the sEMG signal capture, processing, and analysis for the temporalis and masseter muscles. When analyzing the methodological quality, we noted that thirteen of the included studies had low methodological quality, with a high risk of bias. To reduce such a risk of bias, we propose that, in the future, we suggest including the imperative description of the control-group selection method. Moreover, several studies only defined individuals as “with or without TMD.” They did not subdivide individuals according to the classification of the different types of TMD (although a diagnosis was made by widely used validated questionnaires used in research and clinical context). Each kind of TMD may interfere with different parameter changes used to determine masticatory function [55]. On the other hand, we can mention the scarce number of articles available in the literature that used chewing compounds compared with those concerning electromyography. This entails more limited comparability of data and a need for further studies to analyze the chewing function of TMD individuals by chewing compounds. Hence, we can say that, besides the fact that there is a great variety of chewing materials that can be used, few studies fulfilled the inclusion criteria to integrate this present review. The main reasons for these exclusions were an inadequate sample age and a different methodology for TMD diagnosis other than RDC/DC-TMD. This shows the need for future studies to agree on comparable research findings. The chewing-gum methodology is considered to be a suitable method for evaluating chewing patterns, mainly because of the processing easiness and the standardized tests in contrast to natural foods [72]. In the experimental procedure itself, except for one article that assessed laterotrusive movements [37], the dynamic tests were all recorded during habitual chewing or non-habitual chewing (either following a metronome or forced unilateral type). When performed with unilateral or non-habitual mastication, this allows for avoiding possible compensatory adjustments that may arise during contraction of the masticatory muscles, thus obtaining a more stable pattern in muscle recruitment [33]. It also permitted researchers to avoid that the individuals choose their preferred chewing pattern, attributing greater comfort, as happens in habitual chewing [73]. Another limitation verified throughout this systematic review is related to the sample size and diversity of the individuals included in the selected studies. Some of the selected studies had a minimal number of subjects [40,43]. All the studies that evaluated mastication by using the electromyography method used different electromyographs, as well as different frequency domains. The normalization of these parameters would allow for homogenization of the results obtained in those studies.

As for the procedure, we may suggest for future studies a standardization of the methodologies used, either through the electromyography method or with the chewing gum, in order to obtain standardized and comparable results in all the studies carried out.

## 5. Conclusions

Through this review of the literature, we found that the parameters analyzed with the compound technique for chewing showed altered mandibular functions in individuals with TMD. With the EMG method, it was possible to suggest that TMD in adult individuals causes compensatory muscle behaviors. Multiple modifications of the masticatory function were reported, including an asymmetrical and lesser synergy pattern of muscle contractions compared to individuals without TMD. However, it is important to note that a clear association between TMD and chewing disorders could not be determined categorically. Several factors, including sample selection, subjects’ clinical conditions, and research techniques, are particularly important in explaining it.

## Figures and Tables

**Table 1:** PECO Acronym.

P	Population	Adult Individuals (>18 Years) Diagnosed with TMD
E	Exposure	Not applicable
C	Comparators	Masticatory function in individuals without TMD
O	Outcomes	Masticatory function evaluated through: Surface electromyography of the masticatory muscles: electrical activity, frequency, asymmetry of the masticatory muscles Chewing materials: duration of the chewing cycle, chewing efficiency, N° of chewing strokes

**Table 2:** Descriptive characteristics of the studies included.

Chewing Material							
**Reference**	Study Design	Sample	Age	Diagnostic Method	Chewing Evaluation Method	Variables Analysed	Results
[31]	Cross-sectional study	With TMD: 27 individuals (22 F and 5 M) Control: 25 individuals (18 F and 7 M)	TMD: 35.7 ± 9.8 years Control: 30.4 ± 11.5 years	RDC	Capsules with fuscin	Chewing efficiency No. of chewing strokes Chewing time	TMD patients showed greater masticatory efficiency and a higher number of masticatory strokes compared to healthy patients, as well as a modified masticatory pattern, but without compromising the masticatory function
[32]	Cross-sectional study	With TMD: 40 individuals Control: 20 individuals (total: 48 F and 12 M)	20–55 years Mean age: 28 years	RDC	Gelatine cubes	Chewing efficiency	Decreased masticatory efficiency in TMD individuals
[33]	Cross-sectional study	With TMD: 46 subjects (female only) Control: 30 individuals (female only)	TMD: 33.7 ± 11.0 years Control: 29.2 ± 8.9 years	RDC	Cookie	Score total OMES	Lower OMES in TMD
[34]	Cross-sectional study	With TMD: 23 individuals (19 F and 4 M) Control: 23 individuals (18 F and 5 M)	TMD: 28.7 ± 6.2 years Control: 25.5 ± 4.8 years	RDC	Cookie	Score total OMES	Lower OMES in TMD
[35]	Cross-sectional study	With TMD: 42 individuals (female only) Control: 18 individuals (female only)	TMD: 30 ± 8.0 years Control: 26 ± 6 years	RDC	Cookie	Score total OMES	Lower OMES in TMD
[36]	Cross-sectional study	With TMD: 33 individuals (29 F and 4 M) Control: 32 individuals (26 F and 6 M)	TMD: 30.5 ± 7.3 years Control: 28.3 ± 5.8 years	DC	Cookie	Score total OMES	Lower OMES in TMD
**Electromiography**							
**Reference**	**Study** **Design**	**Sample**	**Age**	**Diagnostic Method**	**Chewing Evaluation Method**	**Variables Analysed**	**Results**
[37]	Cross-sectional study	With TMD: 64 individuals (female only) Control: 40 individuals (female only)	TMD: 35.8 ± 15.2 years Control: 35.8 ± 15.2 years	DC	- In Static: MVC + MVC on a COT- In Dynamic: unilateral chewing on right and left side	- In static: POC, TC, IMPACT, ASIM, BAR - In dynamic: SMI	- The only measurements found with statistically significant differences between the 2 groups were BAR and SMI - BAR: Most TMD subjects had measurements outside the reference with the center of gravity positioned anteriorly (temporally predominant) while all healthy subjects had measurements within the reference. - SMI: Most TMD subjects had measurements outside the reference measurements and conversely, control subjects had their measurements within the normal range.
[38]	Cross-sectional study	With TMD: 30 individuals (41 M and 4 H) Control: 15 individuals (14 F and 1 M)	TMD: 25–40 years Control: 27 years	RDC	-In Static: MVC - In dynamic: unilateral chewing on right and left side with pre-softened sugar-free chewing gum	- Static: COTt, COTm, POCt, POCm, POCtors, POCtm, asynergic index - Dynamic: Chewing frequency, Functional index, SMI, Global activity, Activity per cycle	- In Static: TMD subjects had lower masseter and temporalis activity; greater asymmetry of the temporalis muscle compared to the control group; these 2 muscles were also found to be less coordinated in dysfunctional patients. - In dynamic: Similar global activity and the masticatory frequency between the 2 groups; increased SMI; lower functional index in the TMD.
[39]	Cross-sectional study	With TMD: 74 individuals (female only) Control: 30 individuals (female only)	With TMD: 26.54 ± 2.45 years Control: 25.85 ± 2.57 years	RDC	In static: MVC in a parafilm	- MPF: - IEMG	- Electrical activity was significantly lower in the masseter muscles when compared to the anterior temporalis muscles in the TMD group - Media frequency did not change with or without TMD,
[40]	Cross-sectional study	With TMD: 14 individuals (female only) Control: 8 individuals (female only)	With TMD: 28.5 ± 8.6 years Control: 24.7 ± 3.5 years	RDC	In static: MVC in a parafilm	- MF - Fatigue Index	In individuals with TMD: - Fatigue indices were higher compared to controls
[41]	Cross-sectional study	With TMD: 26 individuals (female only) Control: 23 individuals (female only)	TMD: 23.58 ± 3.85 years Control: 21.65 ± 2.76 years	RDC	In static: MVC on a parafilm and rest	- Amplitude - Average frequency MDF	In TMD patients: - In MVC: decreased amplitude, the masseter muscle has a lower average frequency. - At rest: Similar frequency and amplitude
[42]	Cross-sectional study	With TMD: 28 individuals (female only) Control: 27 individuals (female only)	With TMD: 23.50 ± 3.83 years Control: 21.41 ± 2.66 years	RDC	- In Dynamic: During chewing (active and inactive period)	- Symmetry index - APC	In individuals with TMD: - The anterior temporalis muscle is the only muscle studied to show greater asymmetry
[31]	Cross-sectional study	With TMD: 27 individuals (22 F and 5 M) Control: 25 individuals (18 F and 7 M)	TMD: 35.7 ± 9.8 years Control: 30.4 ± 11.5 years	RDC	- In Dynamic: During unilateral chewing, on the right/left side with chewing gum	Average muscle activity	Individuals with TMD: - Less balance in the use of muscles: the anterior temporalis is more solicited than the masseter- However, muscle activity is higher in the TMD group than in the control
[43]	Cross-sectional study	With TMD: 50 individuals (female only) Control: 50 individuals (female only)	TMD: 25–38 years Control: 26–40 years	DC	Static: at rest and during MVC	- Amplitude - Frequency index (during MVC)	TMD subjects: - At rest: increased amplitude parameter was found for both muscles, more in the masseter than in the temporalis compared to the healthy ones on the symptomatic side. - In MVC: decreased amplitude parameter was found for both muscles, more in the temporalis than in the masseter associated with a decrease in frequency
[44]	Cross-sectional study	With TMD: 19 individuals (13 F and 6 M) Control: 19 individuals (13 F and 6 M)	TMD: 25.4 ± 3.8 years Control: 24.1 ± 3.6 years	RDC	At static - At rest - During MVC (maximum voluntary effort) in a parafilm	Muscle activity	Similar values of muscle activity between the two groups
[45]	Cross-sectional study	With TMD: 27 individuals (female only) Control: 18 individuals (female only)	TMD: 38.3 ± 12.8 years Control: 36.2 ± 12.9 years	DC	- In Static: MVC - In dynamic: unilateral chewing on right and left side	- In static: POC, TC, IMPACT, ATTIV - In dynamic: FREQ	TMD subjects: - In static: Similar asymmetric contraction patterns of the masseter and temporalis muscles with the control group; Increased electrical activity of the masseter muscle; Increased muscle work - In dynamic: Chewing frequency and torque similar to control group
[46]	Observational case-control study	With TMD: 30 individuals (female only) Control: 30 individuals (female only)	TMD: 27 ± 7.77 years Control: 23.2 ± 3.78 years	DC	Static: MVC and rest	In static: amplitude, SI, APC	TMD subjects: - In MVC: TMD patients have a smaller amplitude in the right temporalis and left masseter compared to healthy ones - At rest: The EMG values of the masticatory muscles are not modificated by TMD - TMD patients had a greater masseter asymmetry and greater asynergy between muscle pairs
[47]	Observational case-control study	With TMD: 27 individuals (female only) Control: 27 individuals (female only)	TMD: 23.2 ± 4 years Control: 26.4 ± 7.4 years	DC	In dynamic: Habitual and Non-habitual chewing In parafilm	In dynamic: Amplitude, SI, TC, APC	Individuals with TMD: - Reduced activation of the right masseter during the agonist phase in habitual chewing - During the agonist phase, all muscles show a higher activation during non-habitual chewing - Temporal symmetry and APC were decreased during habitual chewing - TC was increased during habitual chewing
[36]	Cross-sectional study	With TMD: 33 individuals (29 F and 4 M) Control: 32 individuals (26 F and 6 M)	TMD: 30.5 ± 7.3 years Control: 28.3 ± 5.8 years	DC	- In Static: MVC - In dynamic: unilateral chewing with pre-softened sugarless gum	- In static: muscle activity - In dynamic: duration of masticatory cycle, relative energy	Individuals with TMD: - In static: decreased muscle activity - In dynamic: increased duration of the masticatory cycle, increased relative energy required to perform the masticatory function
[48]	Observational case-control study	With TMD: 28 individuals (female only) Control: 27 individuals (female only)	TMD and Control: between the ages of 18 and 30	RDC	- In Static: MVC and rest in parafilm	Activation amplitude of temporalis and masseter muscles (muscle electrical activity)	- There was no difference between subjects with and without temporomandibular dysfunction.
[49]	Cross-sectional study	With TMD: 20 individuals Control: 17 individuals M: 29.7% F: 70.3%	TMD: average 40.6 years Control: average 30.2 years	DC	- In Static: MVC and rest	In Static: muscular activity	Individuals with TMD: - In static: decreased values were found in the right and left masseter, and right temporalis compared to control - At rest: Similar muscle activity in TMD
[50]	Cross-sectional study	With TMD: 13 individuals Control: 12 individuals	Between 18 and 40 years	RDC	- In Static: MVC - In dynamic: Lateral movements	Temporal and masseter muscle activity	There was no significant difference between the two groups
[51]	Cross-sectional study	With TMD: 14 individuals (female only) Control: 8 individuals (female only)	TMD: 28.5 ± 8.6 years Control: 24.7 ± 3.5 years	RDC	In dynamic: Bilateral chewing on a parafilm	Total activation times	- There was no significant difference between the two groups
[33]	Cross-sectional study	With TMD: 46 individuals (female only) Control: 30 individuals (female only)	TMD: 33.7 ± 11.0 years Control: 29.2 ± 8.9 years	RDC	In dynamic: one-sided chewing with pre-softened sugar-free gum	- Chewing frequency - amplitude - phase - confidence ellipse - Global activity - Activity per cycle - Symmetrical chewing index	Individuals with TMD: - Chewing frequency and amplitude similar between the groups - Alteration of the coordination between the masseter and temporalis muscles in the working side - Increase of the global activity and per cycle - Decrease of the symmetrical mastication index (SMI)
[35]	Cross-sectional study	With TMD: 42 individuals (female only) Control: 18 individuals (female only)	TMD: 30 ± 8.0 years Control: 26 ± 6 years	RDC	In static: MVC	- POC (T, M), - TC - Muscle activity	Individuals with TMD: - They showed greater asymmetry between the right and left muscle pairs - Unbalanced muscle contractions with the masseter and temporalis muscles control lateralis - Similar muscular activity
[52]	Cross-sectional study	With TMD: 15 individuals (14 F and 1 M) Control: 13 individuals (11 F and 2 M)	TMD: 27.6 ± 7.1 years Control: 28.6 ± 7.0 years	RDC	In static: MVC	Frequency Average, amplitude	Individuals with TMD: - Greater amplitude than the control

ATTIV, Activity Index; BAR, muscular center of gravity. FREQ, Frequency índex; IMPACT, total standardized muscle activity; MVC, maximum voluntary clench; COT, cotton rolls; POC, percentage overlapping coefficient; M, massester muscle; T, temporalis muscle; TC, torque coefficient; SI, symmetry index; SMI, symmetrical mastication index; FI, functional index.

**Table 3:** Summary of the result of the methodological quality assessment of the included studies.

Article	Domain			Conclusions
	Selection	Comparability	Validity	
Kümbüloğlu et al., 2013 [54]	**	**	*	Poor quality
Ferreira et al., 2014 [55]	***	**	*	Good quality
Marim et al., 2019 [56]	****	**	*	Good quality
De Felício et al., 2012 [31]	**	**	*	Poor quality
Di Giacomo et al., 2020 [32]	****	**	*	Good quality
Mapelli et al., 2016 [38]	**	**	*	Poor quality
Pires et al., 2017 [39]	**	**	*	Poor quality
Pitta et al., 2015 [40]	**	**	*	Poor quality
Ries et al., 2016 [41]	***	**	*	Good quality
Ries et al., 2014 [42]	**	**	*	Poor quality
Rodrigues et al., 2015 [44]	**	**	*	Poor quality
Sójka et al., 2018 [48]	**	**	*	Poor quality
Strini et al., 2013 [50]	****	**	*	Good quality
Valentino et al., 2021 [51]	****	**	*	Good quality
Fassicollo et al., 2019 [52]	****	**	*	Good quality
Fassicollo et al., 2019 [33]	****	**	*	Good quality
Fassicollo et al., 2021 [35]	****	**	*	Good quality
Fassicollo et al., 2017 [34]	***	**	*	Good quality
Helena et al., 2021 [36]	**	**	*	Poor quality
Karakis et al., 2021 [37]	**	**	*	Poor quality
Machado et al., 2014 [43]	**	**	*	Poor quality
Xu et al., 2017 [45]	**	**	*	Poor quality

Quality score: Overall scores were given (good, fair, and poor). Good quality: 3 or 4 stars (*) in the selection domain AND 1 or 2 stars in the comparability domain and 2 or 3 stars in the outcome domain; Fair quality: 2 stars in the selection domain and 1 or 2 stars in the comparability domain and 2 or 3 stars in the outcome/exposure domain; poor quality: 0 or 1 star in the selection domain OR 0 stars in the comparability domain OR 0 or 1 stars in the outcome/exposure domain.

**Table 4:** Summaries of studies included in present review.

	Results Difference between Groups YES (+) or NO (−)
Rodrigues et al., 2015 [44]	Chewing efficiency: + No. of chewing strokes: + Chewing time: +
Kümbüloğlu et al., 2013 [54]	Chewing efficiency: +
Ferreira et al., 2014 [55]	Score total OMES: +
Marim et al., 2019 [56]	Score total OMES: +
De Felício et al., 2012 [31]	Score total OMES: +
Fassicollo et al., 2021 [35]	Score total OMES: +
Di Giacomo et al., 2020 [32]	Static: POC: − TC: − IMPACT: − ASSIM: − BAR: + Dynamic: SMI: +
Mapelli et al., 2016 [38]	Static: COTt: + COTm: + POCt: + POCm: − POCtors: − POCtm: + Asynergic index: + Dynamic: Chewing frequency: − Functional Index: + SMI: + Global activity: − Activity per cycle: −
Pires et al., 2017 [39]	MPF: − IEMG: +
Pitta et al., 2015 [40]	MF: − Fatigue Index: +
Ries et al., 2016 [41]	MVC: Amplitude: − Average frequency MF: + At rest: Amplitude: − Average frequency MF: −
Ries et al., 2014 [42]	Symmetry index of T: + Symmetry index of M: − APC: −
Rodrigues et al., 2015 [44]	EMG activity (RM,LM,RT,LT): +
Sójka et al., 2018 [48]	Rest: Amplitude T: + Amplitude M: + MVC: Amplitude: + Frequency index: +
Strini et al., 2013 [50]	Muscle activity: −
Valentino et al., 2021 [51]	Static: POC TA: − POC MM: − TC: − IMPACT: + ATTIV: + Dynamic: FREQ: −
Fassicollo et al., 2019 [52]	MVC: RMS RT: + RMS LT: − RMS RM: − RMS LM: + SI (T): − SI (M): + APC: + At rest: RMS RT: − RMS LT: − RMS RM: + RMS LM: − SI (T): + SI (M): − APC: − APC: −
Fassicollo et al., 2019 [33]	Dynamic: Habitual mastication Amplitude: + only LT SI: − TC: − APC: − Non-habitual mastication Amplitude: − SI: − TC: − APC: −
Fassicollo et al., 2021 [35]	Static: muscle activity: + Dynamic: Duration of masticatory cycle: + Relative energy: +
Fassicollo et al., 2017 [34]	Amplitude: -
Helena et al., 2021 [36]	Static: Muscular activity: + (RM,LM,RT) Rest: Muscular activity: −
Karakis et al., 2021 [37]	Muscular activity: −
Machado et al., 2014 [43]	Total activation times: −
Ferreira et al., 2014 [55]	Chewing frequency: − Amplitude: − Phase: + Confidence ellipse: − Global activity: + Activity per cycle: + SMI: +
De Felício et al., 2012 [31]	POC (T, M): + TC: + Muscle activity: −
Xu et al., 2017 [45]	Amplitude: + only RT MBF: −

## Appendix A. Cochrane Library

**Table A1:** The search strategy used in the Cochrane Library.

Date of Last Survey: 16 June 2022 06:54:34		
#1	Mesh descriptor: [temporomandibular] explode all trees	1874
#2	«Disorder» (word variations were searched)	90,869
#3	#1 and #2	609
#4	Electromyography	6447
#5	«Mastication» (word variations were searched)	911
#6	(chewing)	2682
#7	#5 OR #6	3101
#8	#3 AND #4 AND #7	11
#9	bite force	312
#10	masticatory efficiency	106
#11	mastication cycle	32
#12	#9 OR #10 OR #11	436
#13	#3 AND #12	20
#14	#8 OR #13	29

## Appendix B. PubMed

**Table A2:** The search strategy used on PubMed.

**Last Research Date: 16 June 2022**			
1	temporomandibular	“temporomandibular” [All Fields]	30,251
2	disorder	“disease” [MeSH Terms] OR “disease” [All Fields] OR “disorder” [All Fields] OR “disorders” [All Fields] OR “disorder s” [All Fields] OR “disordes” [All Fields]	64,562
3	(#1) AND (#2)	“temporomandibular” [All Fields] AND “disorder” [All Fields]	18,224
4	electromyography	“electromyography” [MeSH Terms] OR “electromyography” [All Fields] OR “electromyographies” [All Fields]	90,672
5	(mastication) OR (chewing)	“masticated” [All Fields] OR “masticates” [All Fields] OR “masticating” [All Fields] OR “mastication” [MeSH Terms] OR “mastication” [All Fields] OR “masticate” [All Fields] OR “mastications” [All Fields] OR “masticator” [All Fields] OR “chewings” [All Fields] OR “chews” [All Fields] OR “mastication” [MeSH Terms] OR “mastication” [All Fields] OR “chewed” [All Fields] OR “chewing” [All Fields]	25,572
6	((#3) AND (#4)) AND (#5)	“temporomandibular” [All Fields] AND “disorder” [All Fields] AND (“electromyography” [MeSH Terms] OR “mastication” [MeSH Terms] OR “chewing” [All Fields]))	137
7	bite force	“bite force” [MeSH Terms] OR (“bite” [All Fields] AND “force” [All Fields]) OR “bite force” [All Fields]	5028
8	masticatory efficiency	“masticatory” [All Fields] AND (“efficiences” [All Fields] OR “efficiency” [MeSH Terms] OR “efficiency” [All Fields] OR “efficiencies” [All Fields] OR “efficient” [All Fields] OR “efficiently” [All Fields] OR “efficients” [All Fields])	711
9	mastication cycle	(“masticated” [All Fields] OR “masticates” [All Fields] OR “masticating” [All Fields] OR “mastication” [MeSH Terms] OR “mastication” [All Fields] OR “masticate” [All Fields] OR “mastications” [All Fields] OR “masticator” [All Fields]) AND (“cycle” [All Fields] OR “cycle s” [All Fields] OR “cycled” [All Fields] OR “cycles” [All Fields] OR “cycling” [All Fields] OR “cyclings” [All Fields])	1090
10	((#7) OR (#9)) OR (#10)	“bite force” [MeSH Terms] OR (“masticatory” [All Fields] AND (“efficiences” [All Fields] OR “efficiency” [MeSH Terms] “cycles” [All Fields]	6450
11	(#3) AND (#10)	“temporomandibular” [All Fields] AND “disorder” [All Fields] AND “bite force” [MeSH Terms] OR (“masticatory” [All Fields] AND (“efficiences” [All Fields] OR “efficiency” [MeSH Terms] “cycles” [All Fields]	407
12	(#6) OR (#11)	“temporomandibular” [All Fields] AND “disorder” [All Fields] AND (“electromyography” [MeSH Terms] OR “mastication” [MeSH Terms] OR “chewing” [All Fields])) OR “temporomandibular” [All Fields] AND “disorder” [All Fields] AND “bite force” [MeSH Terms] OR (“masticatory” [All Fields] AND (“efficiences” [All Fields] OR “efficiency” [MeSH Terms] “cycles” [All Fields]	508
13	(#6) OR (#11)	“temporomandibular” [All Fields] AND “disorder” [All Fields] AND (“electromyography” [MeSH Terms] OR “mastication” [MeSH Terms] OR “chewing” [All Fields])) OR “temporomandibular” [All Fields] AND “disorder” [All Fields] AND “bite force” [MeSH Terms] OR (“masticatory” [All Fields] AND (“efficiences” [All Fields] OR “efficiency” [MeSH Terms] “cycles” [All Fields]	234

## Appendix C. EBSCO

**Table A3:** The search strategy used on EBSCO.

#	Consulta	Limitadores/Expansores	última Execução Por	Resultados
S9	S1 AND S8	Limitadores—data de Publicação: 20110101-20211231 Expansores—Aplicar assuntos equivalentes Modos de pesquisa—Booleana/Frase	Interface—EBSCOhost research Databases Ecrã e Pesquisa—Pesquisa Avançada Base de dados—CINAHL with Full Text; Dentistry & Oral Sciences Sources; MEDLINE Complete	259
S8	S1 AND S7	Limitadores—aplicar assuntos equivalentes Modos de pesquisa—Booleana/Frase	Interface—EBSCOhost research Databases Ecrã e Pesquisa—Pesquisa Avançada Base de dados—CINAHL with Full Text; Dentistry & Oral Sciences Sources; MEDLINE Complete	511
S7	Mastication cycle OR masticatory efficiency OR bite force	Limitadores—aplicar assuntos equivalentes Modos de pesquisa—Booleana/Frase	Interface—EBSCOhost research Databases Ecrã e Pesquisa—Pesquisa Avançada Base de dados—CINAHL with Full Text; Dentistry & Oral Sciences Sources; MEDLINE Complete	7073
S6	S4 AND S5	Limitadores—aplicar assuntos equivalentes Modos de pesquisa—Booleana/Frase	Interface—EBSCOhost research Databases Ecrã e Pesquisa—Pesquisa Avançada Base de dados—CINAHL with Full Text; Dentistry & Oral Sciences Sources; MEDLINE Complete	280
S5	S1 AND S2	Limitadores—aplicar assuntos equivalentes Modos de pesquisa—Booleana/Frase	Interface—EBSCOhost research Databases Ecrã e Pesquisa—Pesquisa Avançada Base de dados—CINAHL with Full Text; Dentistry & Oral Sciences Sources; MEDLINE Complete	1032
S4	Mastication OR chewing	Limitadores—aplicar assuntos equivalentes Modos de pesquisa—Booleana/Frase	Interface—EBSCOhost research Databases Ecrã e Pesquisa—Pesquisa Avançada Base de dados—CINAHL with Full Text; Dentistry & Oral Sciences Sources; MEDLINE Complete	37,514
S3	Temporomandibular AND disorder OR electromyography	Limitadores—aplicar assuntos equivalentes Modos de pesquisa—Booleana/Frase	Interface—EBSCOhost research Databases Ecrã e Pesquisa—Pesquisa Avançada Base de dados—CINAHL with Full Text; Dentistry & Oral Sciences Sources; MEDLINE Complete	138,322
S2	electromyography	Limitadores—aplicar assuntos equivalentes Modos de pesquisa—Booleana/Frase	Interface—EBSCOhost research Databases Ecrã e Pesquisa—Pesquisa Avançada Base de dados—CINAHL with Full Text; Dentistry & Oral Sciences Sources; MEDLINE Complete	112,218
S1	Temporomandibular AND disorder	Limitadores—aplicar assuntos equivalentes Modos de pesquisa—Booleana/Frase	Interface—EBSCOhost research Databases Ecrã e Pesquisa—Pesquisa Avançada Base de dados—CINAHL with Full Text; Dentistry & Oral Sciences Sources; MEDLINE Complete	27,136

## Appendix D. Scielo

**Table A4:** The search strategy used on Scielo.

Id.	Busca	Resultados
#11	Expressão: (#6) OR (#8) Filtros aplicados: (Ano de publicação: 2018) (Ano de publicação: 2019) (Ano de publicação: 2017) (Ano de publicação: 2016) (Ano de publicação: 2020 (Ano de publicação: 2015) (Ano de publicação: 2014) (Ano de publicação: 2011)	20
#10	Expressão:(((((temporomandibular) AND (disorder) AND network:org AND network:rve) OR (electromyography AND network:org AND network:rve) AND network:org AND network:rve) AND ((mastication) OR (chewing) AND network:org AND network:rve) AND network:org AND network:rve) OR (((temporomandibular) AND (disorder) AND network:org AND network:rve) AND (bite force) OR (masticatory efficiency) OR (mastication cycle) NAD network:org AND network:rve) AND network:org AND network:rve) AND network:org AND network:rve	1
#9	Expressão: (#6) OR (#8) Filtros aplicados:	39
#8	Expressão: (#1) OR (#7) Filtros aplicados:	1
#7	Expressão: (BITE FORCE) OR (masticatory efficiency) OR (mastication cycle) Filtros aplicados:	109
#6	Expressão: (#4) OR (#5) Filtros aplicados:	38
#5	Expressão: (MASTICATION) OR (CHEWING) Filtros aplicados:	780
#4	Expressão: (#1) OR (#2) Filtros aplicados:	997
#3	Expressão: ( Filtros aplicados:	0
#2	Expressão: ELECTROMYOGRAPHY Filtros aplicados:	759
#1	Expressão:(TEMPOROMANDIBULAR) OR (DISORDER) Filtros aplicados:	259

## Appendix E. ClinicalTrials.gov

**Table A5:** The search strategy used on ClinicalTrials.gov.

Terms Synonyms	Search Results
**Electromyography** Electromyogram	5 studies --
Masticatory efficiency	2 studies
efficiency	4 studies
masticatory	6 studies
**Temporomandibular Disorder** TEMPOROMANDIBULAR JOINT DISORDER Temporomandibular joint dysfunction Temporomandibular dysfunction Temporomandibular Joint Disease Costen Syndrome Dysfunction tmj Mandibular dysfunction Pain-dysfunction syndrome Temporomandibular Joint Syndrome TMJ Disease TMJ Disorder Tmj dysfunction	8 studies 7 studies 7 studies 1 studies 1 studies -- -- -- -- -- -- -- --
**Disorder** Diseases condition	8 studies 8 studies --
**Temporomandibular**	8 studies

## Appendix F. Excluded Articles

**Table A6:** Articles excluded through after applying selection and eligibility.

Articles Excluded-Selection (*n* = 213)	
*n* = 186	Title and/abstract
*n* = 3	Language (Russian)
*n* = 3	Case report
*n* = 5	Literature review
*n* = 14	Systematic review
*n* = 1	Interview
*n* = 1	Free complete text unavailable
**Articles Excluded—Eligibility (*n* = 45)**	
*n* = 15	Inappropriate sample
*n* = 6	Used other TMD diagnostic method than RDC-TMD and DC-TMD
*n* = 3	Without link with TDM/Used other TMD diagnostic method than RDC-TMD and DC-TMD
*n* = 6	Inappropriate intervention
*n* = 12	Without link with TDM
*n* = 2	Used other TMD diagnostic method than RDC-TMD and DC-TMD Inappropriate sample
*n* = 1	Without link with TDM Inappropriate sample

## Appendix G. Description of the Aspects Contained in the Newcastle–Ottawa Quality Assess

### Ment Scale

**Table:**

	Autor/Year
Selection 1—Representativeness of the sample	(a) Truly representative of the average in the target population * (all subjects or random sampling). (b) Somewhat representative of the average in the target population (nonrandom sampling). * (c) Selected group of users. (d) No description of the sampling strategy.
2—Sample size	(a) Justified and satisfactory. * (b) Not justified.
3—Non-respondents	(a) Comparability between respondents’ and non-respondents’ characteristics is established, and the response rate is satisfactory. * (b) The response rate is unsatisfactory, or the comparability between respondents and non-respondents is unsatisfactory. (c) No description of the response rate or the characteristics of the responders and the non-responders.
4—Ascertainment of the exposure (risk factor)	(a) Validated measurement tool. * (b) Non-validated measurement tool, but the tool is available or described. (c) No description of the measurement tool.
Comparability: The subjects in different outcome groups are comparable based on the study design or analysis. Confounding factors are controlled.	(a) Study controls for age. (b) Study controls for gender.
Outcomes 1—Assessment of the outcome	(a) Independent blind assessment. (b) Record linkage. ** (c) Self-report. * (d) No description.
2—Statistical test	(a) The statistical test used to analyze the data is clearly described and appropriate, and the measurement of the association is presented, including confidence intervals and the probability level (*p*-value). * (b) The statistical test is not appropriate, not described, or incomplete.
TOTAL	

According to the Newcastle-Ottawa Scale (NOS) criteria. Quality score: Overall scores were given (good, fair, and poor). Good quality: 3 or 4 stars (*) in the selection domain AND 1 or 2 stars in the comparability domain and 2 or 3 stars in the outcome domain; Fair quality: 2 stars in the selection domain and 1 or 2 stars in the comparability domain and 2 or 3 stars in the outcome/exposure domain; poor quality: 0 or 1 star in the selection domain OR 0 stars in the comparability domain OR 0 or 1 stars in the outcome/exposure domain.
